# Anterolateral Thigh Island Flap Reconstruction After Margin-Positive, Sphincter-Preserving Excision of Scrotal Extramammary Paget’s Disease: A Case Report

**DOI:** 10.3390/jcm15218508

**Published:** 2026-11-02

**Authors:** Jeonghun Ahn, Jin Sil Seo, Kyung Min Kim, Ki Hyun Kim, Jai Koo Choi, In Suck Suh, Seong Hwan Kim

**Affiliations:** Department of Plastic and Reconstructive Surgery, Kangnam Sacred Heart Hospital, College of Medicine, Hallym University, Seoul 07441, Republic of Korea; jeonghun@hallym.or.kr (J.A.); minora1@hallym.or.kr (J.S.S.); baoro333@gmail.com (K.M.K.); rlarlgus0608@hallym.or.kr (K.H.K.); jkchoi1957@hallym.or.kr (J.K.C.); sismdps@hallym.or.kr (I.S.S.)

**Keywords:** Paget disease, extramammary, scrotum, perineum, surgical flaps, margins of excision, case report

## Abstract

**Background/Objectives:** Extramammary Paget’s disease (EMPD) of the scrotum, a rare intraepithelial adenocarcinoma, closely mimics benign dermatoses and is often diagnosed only after years of unsuccessful topical treatment. Because tumor cells extend beyond the visible border, wide excision often leaves large defects and positive margins. When disease reaches the perianal skin, clearance may cost anorectal function. **Case:** A 69-year-old male had been treated for presumed scrotal eczema for 9 years before punch biopsy established EMPD extending from the scrotum across the perineum to the medial thigh and anal verge. After wide excision with a 2-cm margin, frozen section showed intraepidermal Paget cells at the perianal margin; because clearance risked sphincter resection, further excision was withheld. The continuous defect was reconstructed with an 18 × 12 cm anterolateral thigh (ALT) island flap. The flap survived completely. Medical oncology recommended surveillance, with radiotherapy or systemic therapy reserved for progression. The site remained stable at 6 months; the patient was then lost to follow-up untreated. **Conclusions:** When a positive margin is accepted to preserve function, tissue that can be re-elevated, tolerates irradiation, and leaves residual disease accessible to inspection is a reasonable choice, although this remains untested here and the oncologic outcome is unknown.

## 1. Introduction

Extramammary Paget’s disease (EMPD) is an uncommon intraepithelial adenocarcinoma arising in apocrine-rich skin. The vulva is the most frequently affected site, followed by the perianal region. In contrast, cases involving the penis and scrotum together remain limited [1,2]. Male genital involvement is nevertheless reported disproportionately often in East Asian populations [3,4].

The clinical appearance of EMPD—a well-demarcated, erythematous, moist or scaly plaque accompanied by itching—can be difficult to distinguish from eczema, contact dermatitis, or superficial fungal infection. Patients are therefore commonly managed with topical steroids or antifungal agents for long periods. Misdiagnosis at first presentation has been described in most cases [1,5]. Diagnosis requires histologic identification of Paget cells, and immunohistochemistry aids in confirming the diagnosis and distinguishing EMPD from Bowen’s disease and melanoma, which may present with similar clinical features [2,6].

Wide local excision remains the primary treatment. However, Paget cells can spread laterally within the epidermis and adnexal epithelium far beyond the visible lesion, and the disease may be multicentric. Consequently, even a 2-cm clinical margin may leave microscopically positive margins in many patients [7,8]. When the lesion extends to the perineum and perianal skin, the anatomic price of complete clearance may become prohibitive, and the surgeon is forced to weigh oncologic completeness against anorectal function.

Because diagnosis is often delayed, the defect is usually too large for primary closure. Split-thickness skin graft and local scrotal flaps have both been used [9,10]; however, grafts placed on the mobile, convex penoscrotal surface are prone to contracture and incomplete take, and local flaps offer limited options when most scrotal skin has been resected. The anterolateral thigh (ALT) flap provides a large, pliable, well-vascularized skin paddle on a pedicle long enough to reach the perineum as an island flap without the need for microvascular anastomosis [11,12]. Reconstruction of penoscrotal defects with the ALT flap after excision of EMPD has been reported previously; however, the reports we identified were under the premise that the excision was complete, with the flap justified by the size and contour of the defect [11,12]. The technique itself is therefore not new; the present report differs in that a positive margin was accepted intentionally. We describe a patient whose disease extended from the scrotum across the perineum onto the medial thigh and reached the anal verge, in whom a positive perianal margin was accepted to preserve the anal sphincter. Our aim is to propose, with supporting evidence, how a positive margin accepted for functional reasons alters the requirements placed on the reconstruction, and to report the donor-site morbidity encountered.

## 2. Case

**Figure 1:**
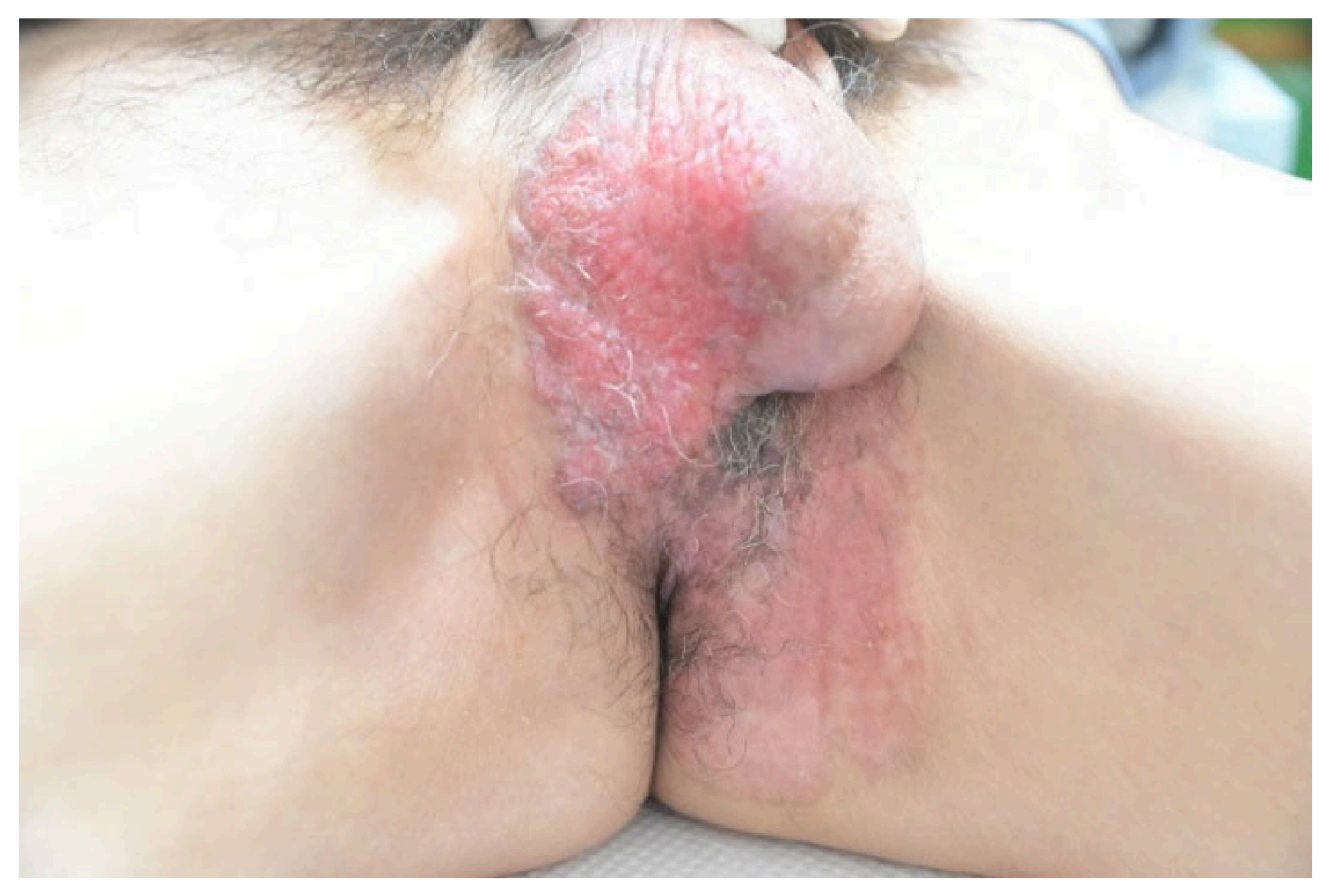
Preoperative appearance of the lesion. A well-demarcated erythematous plaque involved the scrotum and extended across the perineum to the medial aspect of the left proximal thigh (outlined), reaching the immediate vicinity of the anus, after 9 years of treatment for presumed eczema.

**Figure 2:**
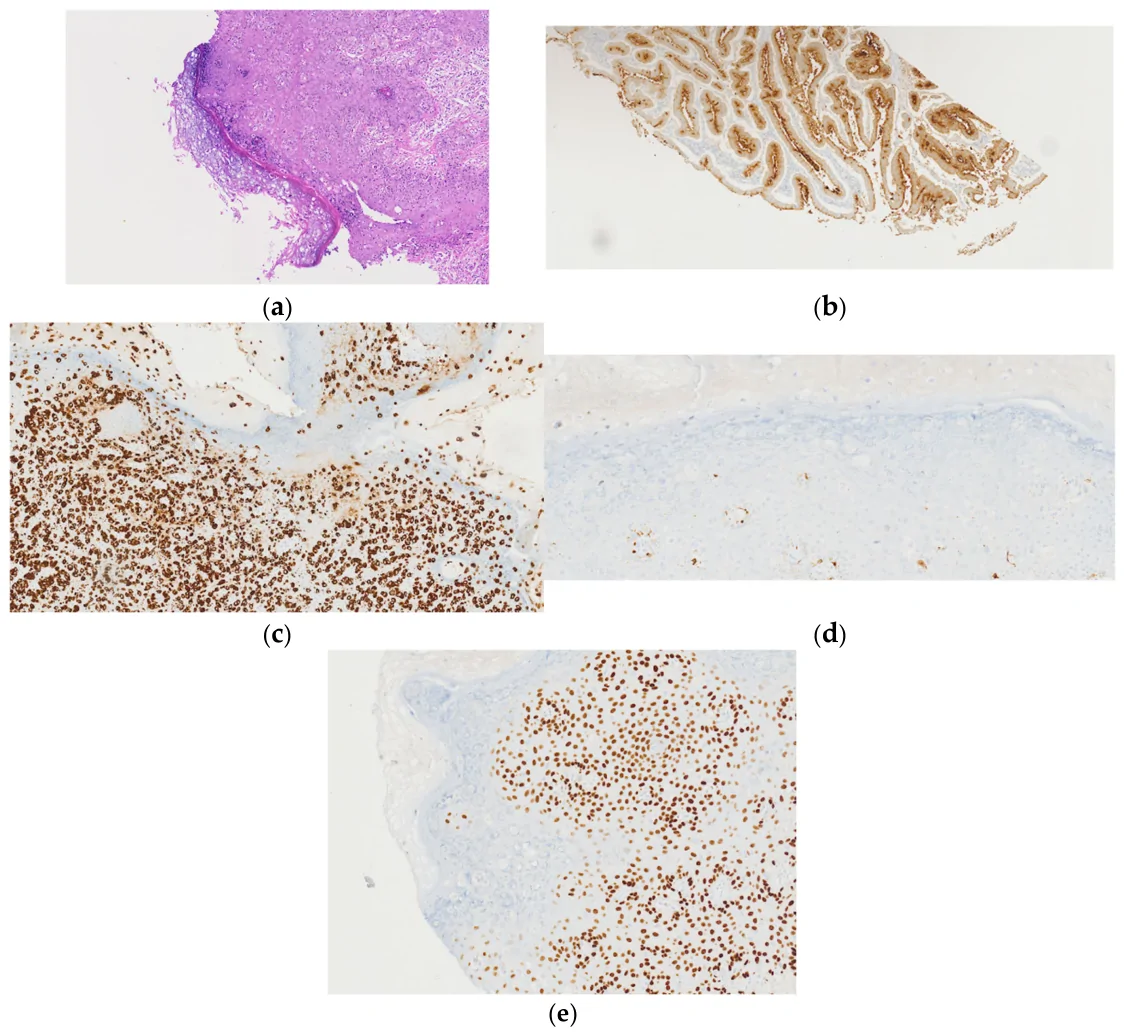
Hematoxylin and eosin (HE) and immunohistochemistry (IHC) stained photomicrographs of the punch biopsy. (**a**) Pale-staining Paget cells within the epidermis, original magnification 5×; (**b**) CEA, original magnification 5× and (**c**) CK7, original magnification 5× show positivity in the Paget cells; (**d**) Melan-A, original magnification 10× and (**e**) p63, original magnification 10× are negative.

A 69-year-old male with hypertension presented with a pruritic erythematous scrotal lesion of approximately 9 years’ duration, treated intermittently as eczema at a local dermatology clinic without improvement. He had smoked 0.5 packs of cigarettes per day for 30 years and did not drink alcohol, and he had no family history of any disease. The plaque was not confined to the scrotum: it extended across the perineum to the medial aspect of the left proximal thigh and reached the immediate vicinity of the anus (Figure 1). No inguinal lymph nodes were palpable. The clinical differential diagnosis included chronic eczema, contact dermatitis, superficial fungal infection, psoriasis, Bowen’s disease, and amelanotic melanoma [4,6,11]. The patient was referred to the department of dermatology in March 2025, where punch biopsy suggested EMPD, and was subsequently transferred to our department for surgical treatment. A repeat punch biopsy in April 2025 confirmed the diagnosis; the tumor cells were positive for carcinoembryonic antigen (CEA) and cytokeratin 7 (CK7) and negative for p63 and Melan-A (Figure 2). The clinical course is summarized in Table 1.

Because the depth of invasion cannot be established from punch biopsies of a large plaque, staging was performed before excision to detect regional nodal or distant metastasis, using contrast-enhanced computed tomography (CT) of the chest and abdominopelvic cavity, contrast-enhanced pelvic magnetic resonance imaging (MRI), and bone scintigraphy, as is routine for patients with cancer at our institution. CT showed a small enhancing nodule, approximately 8 mm in size, in the left scrotum, whereas MRI showed only diffuse thickening of the scrotal wall. No pathological inguinal lymphadenopathy, no evidence of an underlying visceral malignancy, and no distant metastasis were identified. The further investigations recommended to exclude secondary EMPD for this anatomic distribution—colonoscopy, cystoscopy, urine cytology, fecal occult blood testing, and serum prostate-specific antigen (PSA) [13,14]—were not performed, nor was immunohistochemistry for cytokeratin 20 (CK20) and gross cystic disease fluid protein 15 (GCDFP-15); CK7 positivity is consistent with, but does not establish, primary EMPD [15].

**Figure 3:**
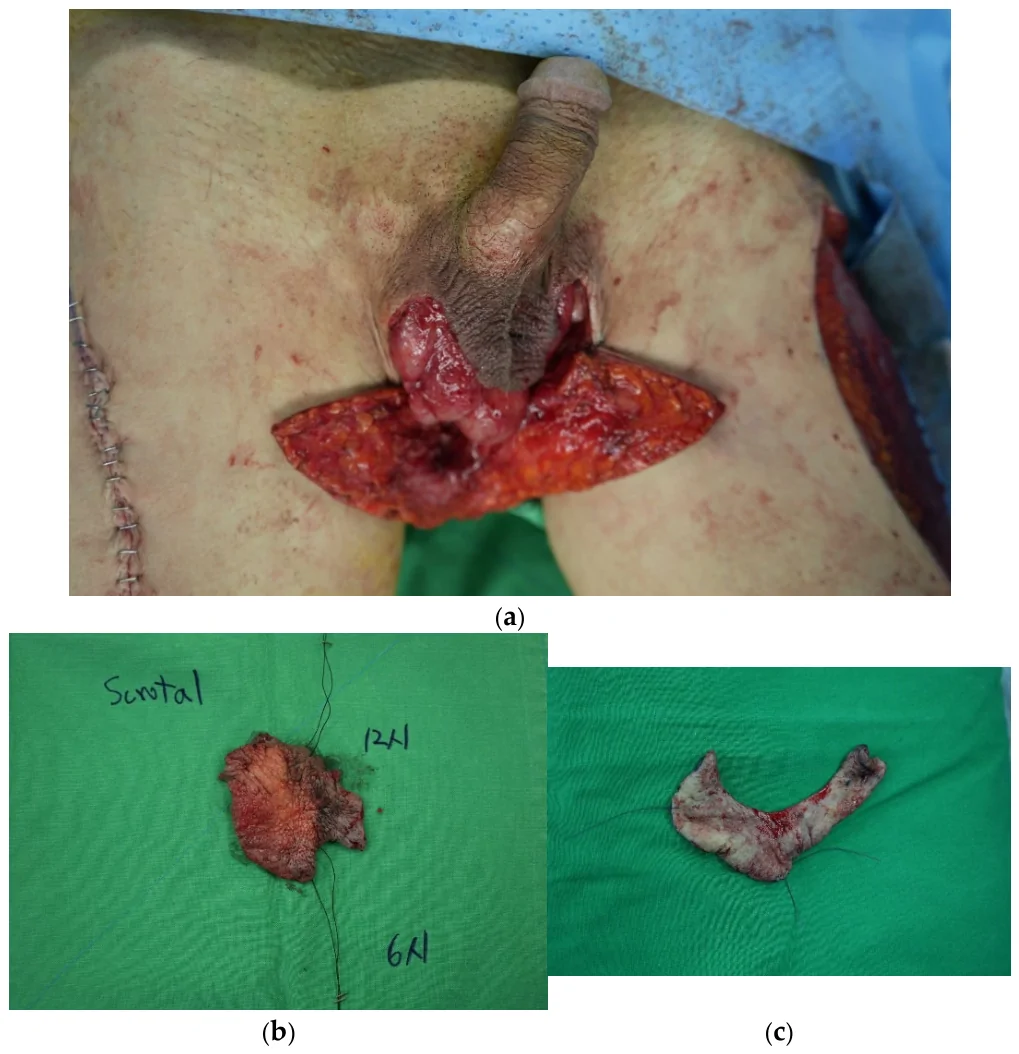
Wide excision of the penoscrotal and perineal lesion. (**a**) The defect after resection. (**b**,**c**) The excised specimens; the handwritten markings in Korean indicate the 12 o’clock and 6 o’clock positions for specimen orientation.

Surgery was performed in May 2025 under general anesthesia. Prior to excision, a single perforator was identified near the midpoint of the line connecting the anterior superior iliac spine and the patella, and an 18 × 12 cm ALT flap was designed on the anterolateral aspect of the left thigh, remote from the medial extension of the disease. The urology team performed wide excision of the scrotal lesion including the nodule in the left scrotum, and our team excised the perineal extension on the medial aspect of the left thigh, with a 2-cm clinical margin around the entire visible lesion. Intraoperative frozen section showed intraepidermal Paget cells at the perianal margin, whereas all other peripheral margins were clear. Additional resection was considered; however, this margin lay at the anal verge, so that clearance would have required excision of the anoderm to an unknown proximal extent and, if the margin remained positive, resection of the anus with permanent loss of continence. After intraoperative consultation with the departments of general surgery and medical oncology, further excision was not performed, because the frozen section showed only intraepidermal disease at this margin and staging had shown no nodal or distant spread, and leaving residual disease that could be treated without further surgery if it progressed was judged preferable to sacrificing the sphincter. The residual disease was thus presumed, but not confirmed, to be intraepidermal, as the retained skin was not sampled; anorectal function was preserved (Figure 3).

**Figure 4:**
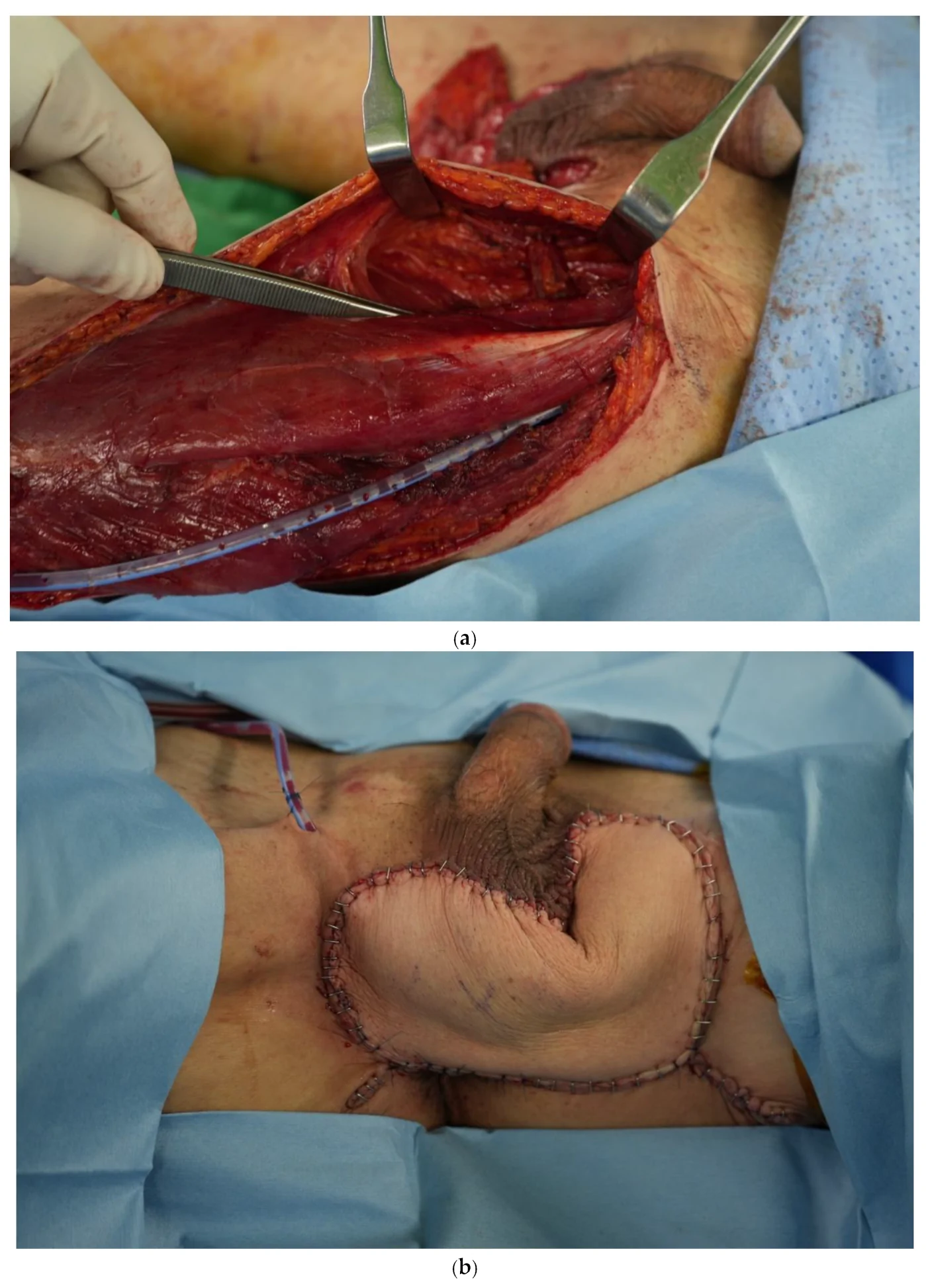
Elevation and inset of the anterolateral thigh island flap. (**a**) An 18 × 12 cm flap designed on the anterolateral aspect of the left thigh over a perforator located near the midpoint between the anterior superior iliac spine and the patella. (**b**) The flap after subcutaneous tunneling and inset.

The flap was elevated as a fasciocutaneous unit including the fascia lata, and the perforator, which ran a septocutaneous course through the intermuscular septum without intramuscular dissection, was traced proximally to the descending branch of the lateral circumflex femoral artery, yielding a pedicle approximately 12 cm in length. Islanded on this pedicle, the flap was tunneled subcutaneously into the perineum and inset to resurface the penoscrotal, perineal, and medial thigh defect as a single unit. The skin of the flap was used to envelop the testes and reconstitute the scrotal sac, and the flap was secured so that no tension was transmitted to the pedicle. An audible Doppler signal and normal capillary refill were confirmed after inset (Figure 4).

**Figure 5:**
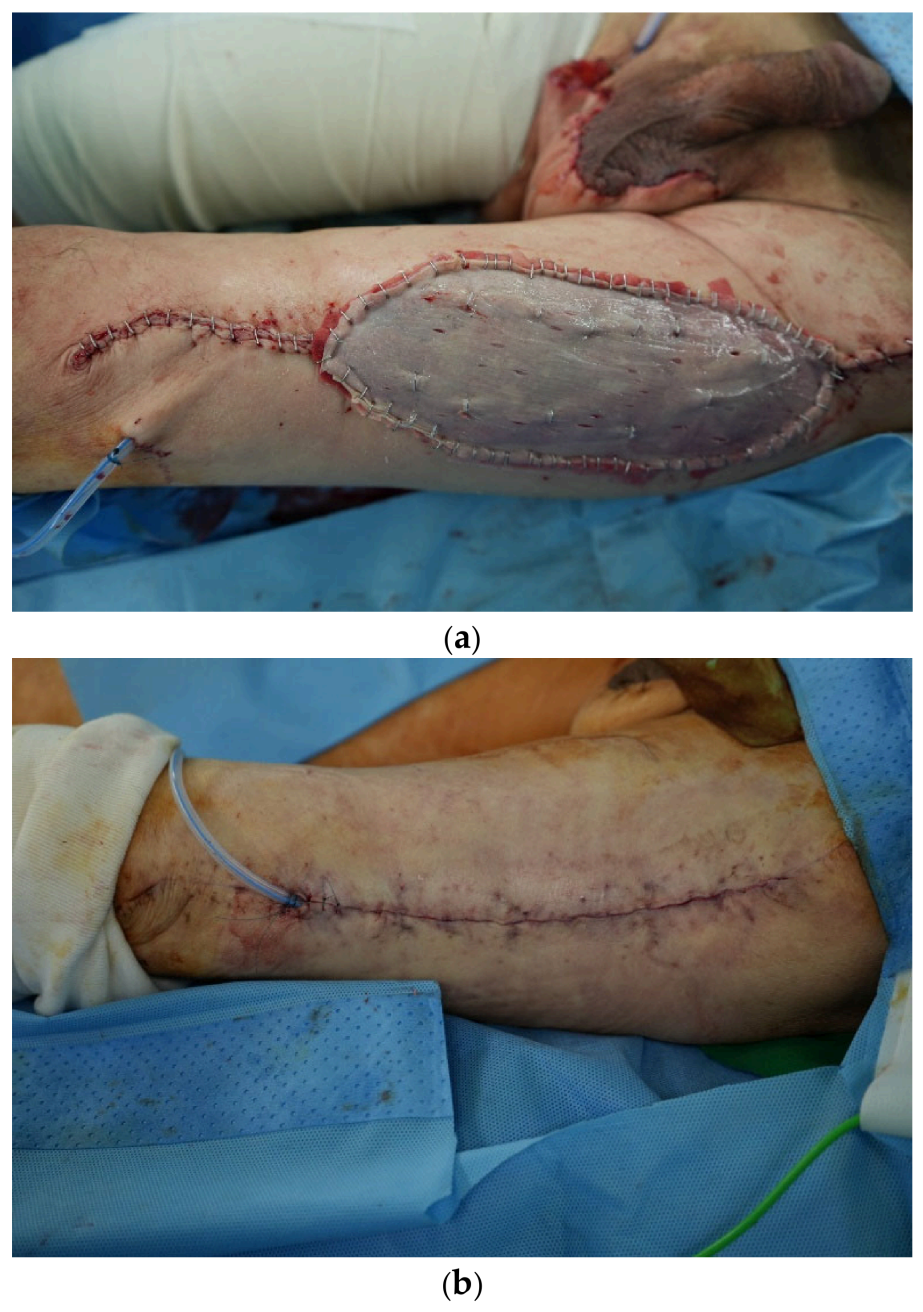
Course of the donor site. (**a**) Coverage with an acellular dermal matrix and a split-thickness skin graft harvested from the right thigh. (**b**) Local advancement of skin and underlying muscle on postoperative day 19.

The donor-site defect was too wide for primary closure. Trial approximation of the wound edges compressed the pedicle and risked venous congestion of the flap, so primary closure was abandoned. Acellular dermal matrix was applied over the exposed vastus lateralis to promote adherence and limit contracture, and was covered with a split-thickness skin graft harvested from the right thigh (Figure 5a). Postoperatively, the patient was kept on bed rest for 1 week with the hips held in a frog-leg position to limit tension and shear at the perineal inset, and an indwelling urinary catheter was maintained for 2 weeks.

**Figure 6:**
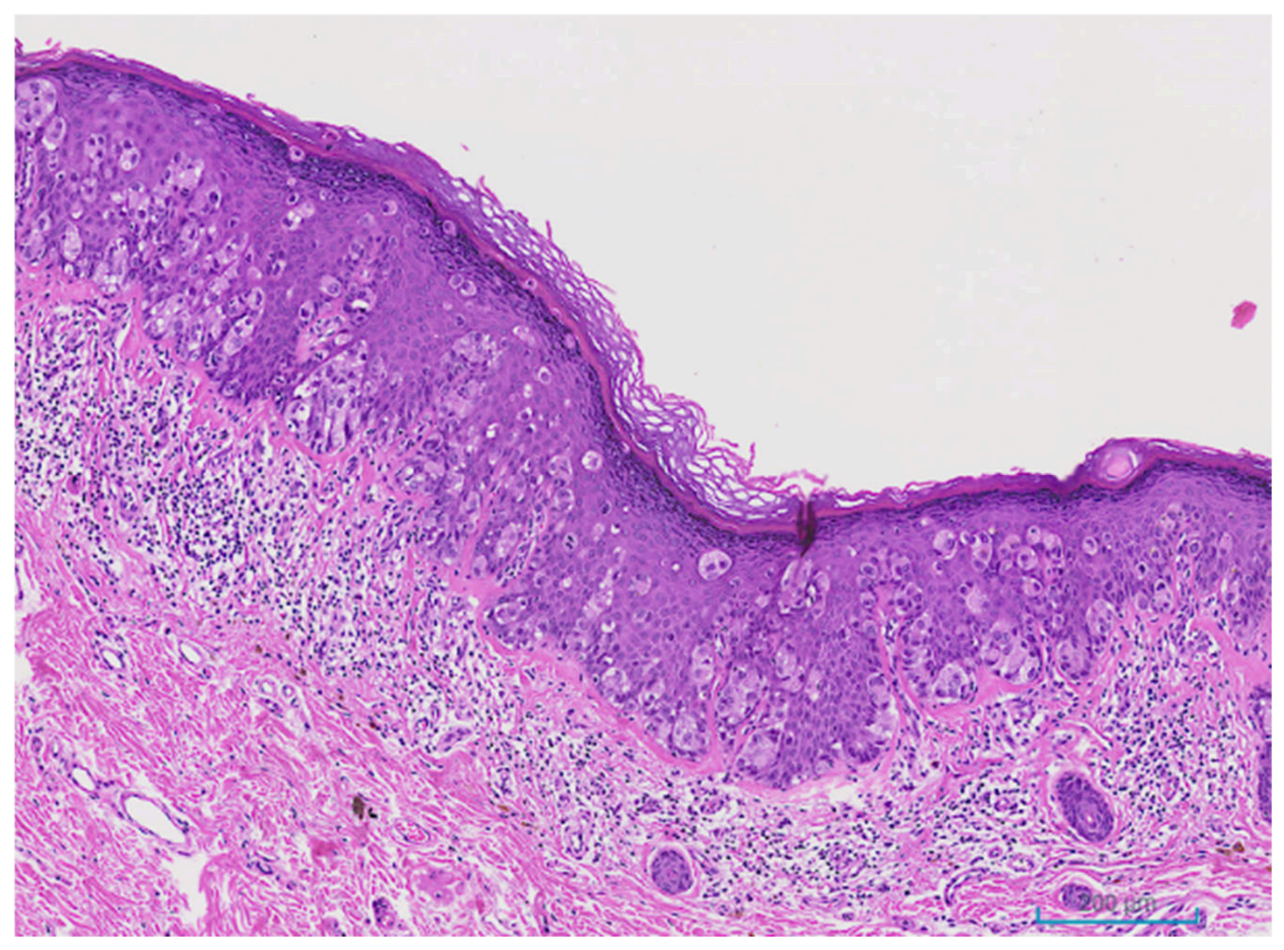
Photomicrograph of the resection specimen, original magnification 5×. Paget cells are seen within the epidermis and adnexal epithelium, with no invasion of the dermis.

The flap survived completely; however, the graft over the donor site failed to take, leaving a 15 × 8 cm defect. On postoperative day 19, under local anesthesia, the wound was irrigated and the margins were debrided until clear. As the defect was too wide for direct closure without risk of dehiscence, the surrounding muscle and soft tissue were dissected for approximately 2 cm and advanced together with the overlying skin; approximation of the muscle and skin flaps produced no contour depression, and the wound was closed (Figure 5b). The patient was discharged on postoperative day 22. Permanent pathology confirmed EMPD confined to the epidermis and adnexal epithelium, with no dermal invasion and no lymphovascular or perineural invasion (Figure 6). The deep margin and all peripheral margins were clear except the perianal margin, where intraepidermal Paget cells were present, confirming the frozen-section finding at the margin that had been accepted intraoperatively. HER2 immunostaining was not performed.

**Figure 7:**
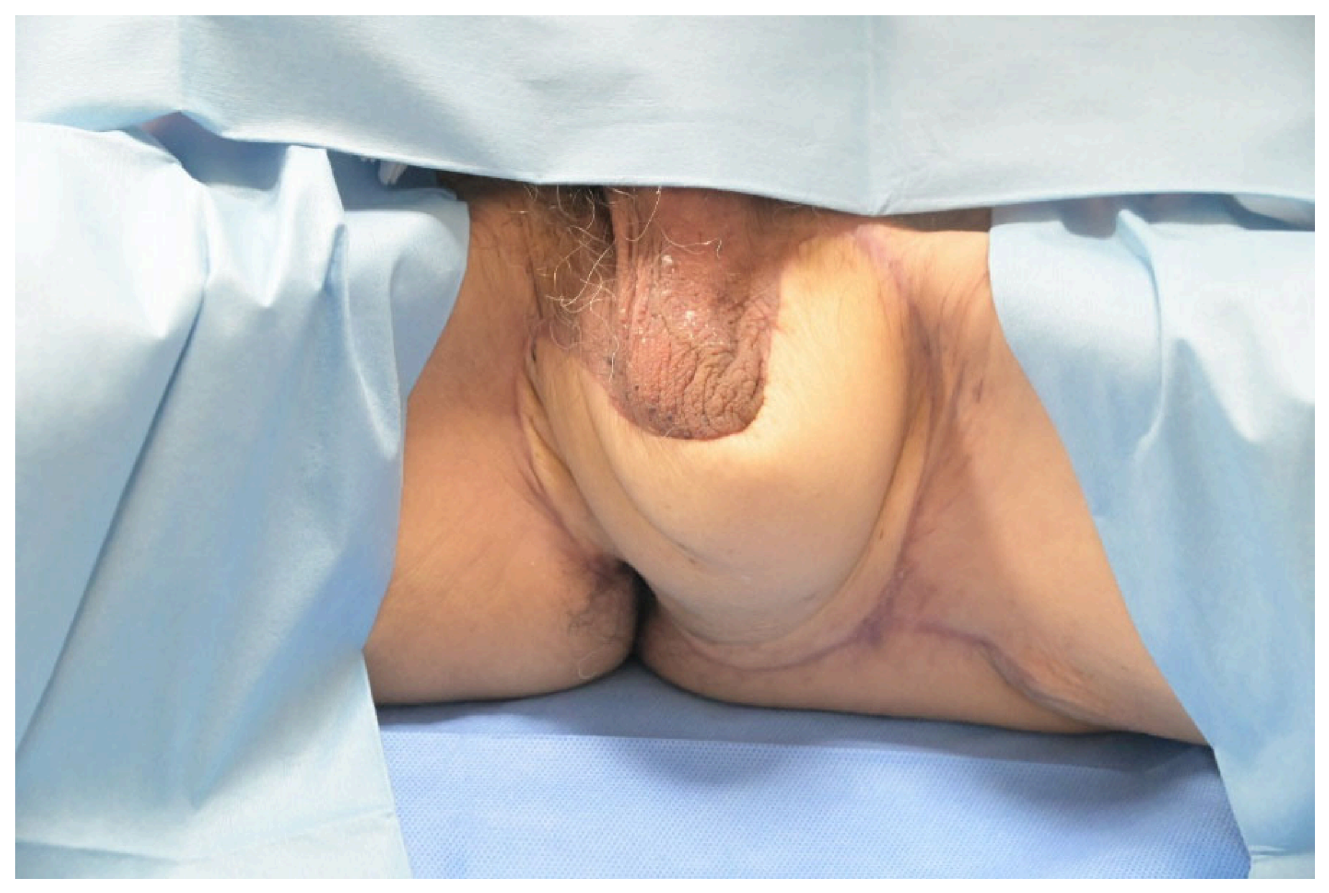
Appearance at the last outpatient visit, 6 months after discharge. The reconstructed scrotum and the thigh donor site had healed without clinically apparent recurrence.

At the medical oncology visit 1 month after discharge, surveillance was recommended, with radiotherapy or systemic therapy to be started if the residual disease progressed, in view of the slowly progressive course of intraepidermal EMPD; no specific modality, regimen, dose, or schedule was defined. The need for continued surveillance, and for further treatment should the residual disease progress, was explained to the patient. He was reviewed in the outpatient clinic 5 days, 2 weeks, 1 month, 3 months, and 6 months after discharge; at each visit, the reconstructed area and the perianal skin were examined, no suspicious change was found, and no biopsy was taken. Because no progression was observed, no further treatment was started. At the last outpatient visit, 6 months after discharge, the reconstructed scrotum and the thigh had healed and no clinically apparent recurrence was identified (Figure 7). Defecation and voiding were normal without incontinence, he walked without difficulty, hip and knee motion was unrestricted, and there was no donor-site pain; sexual function was not assessed. The patient did not visit thereafter and was lost to follow-up; attempts to contact him were unsuccessful, no further treatment was delivered, and a patient perspective could not be obtained.

## 3. Discussion

The 9-year interval between symptom onset and diagnosis in our patient was longer than the 2–4 years usually reported for EMPD; however, it reflects the same mechanism: a chronic, itchy genital plaque is treated empirically as dermatitis, and each partial response to topical steroids postpones biopsy [1,5]. Any eczematous genital lesion that fails to resolve within a few weeks of appropriate topical therapy should be biopsied. Delay enlarges both the lesion and the field of subclinical spread; here the plaque had crept from the scrotum across the perineum to the anal verge, an extent consistent with the lateral intraepidermal migration described for EMPD [8]. Because Paget cells advance beyond the erythematous border, microscopically positive margins follow a conventional 2-cm clinical margin in about 40% of penoscrotal cases, and recurrence correlates closely with margin status [7,8]. Frozen-section guidance and Mohs micrographic surgery lower this rate but demand operative time and pathology resources, and neither reliably samples a discontinuous advancing front [16].

The positive margin in our patient was of a different kind. Its location was identified intraoperatively by frozen section, and it was accepted deliberately, because pursuing clearance at the anal verge might ultimately have required sacrifice of the anal sphincter complex; comparable dilemmas are described in perianal Paget’s disease [17]. Three considerations supported this decision. First, the frozen section showed only intraepidermal disease at that margin, all other margins were clear, and preoperative staging had shown no nodal or distant disease. Second, intraepidermal EMPD progresses slowly, and current guidelines list radiotherapy, topical imiquimod, and photodynamic therapy as alternatives for intraepidermal disease that cannot be resected [13]. Third, leaving a positive margin at a critical structure to preserve function is an established approach in soft tissue sarcoma surgery, where such margins carry a lower risk of local recurrence than unexpected positive margins [18]. Such a decision should not rest with the resecting surgeon alone. In our case, intraoperative consultation with general surgery and medical oncology made the trade-off explicit—permanent loss of anorectal function against residual intraepidermal disease that could be treated without further surgery if it progressed. The main weakness of this decision is that the retained skin was not sampled, either during surgery or afterwards; the presumption that the residual disease was intraepidermal rests on the margin specimen alone, and occult invasion in the retained field cannot be excluded.

We therefore propose that such a decision, once taken, should govern the reconstruction rather than follow from it. Reports of flap reconstruction after excision of penoscrotal EMPD generally assume that clear margins have been achieved, with flap selection driven by the size and contour of the defect [11,12]. When disease is knowingly retained, three further requirements can be proposed, each derived from published evidence rather than from the outcome of this case. First, the reconstruction should be re-elevatable, because recurrence after excision of EMPD is frequent [7,8] and surgery offers the highest reported cure rates [13]; a flap can be re-elevated more readily than a skin graft healed onto granulation tissue [11]. Second, the reconstructed tissue should tolerate subsequent irradiation, because radiotherapy is a recognized non-surgical option for residual EMPD [13,19]. Flap reconstruction of the previously irradiated perineum has reduced major wound complications compared with primary closure [20], and flaps irradiated after transfer also fare well: postoperative radiotherapy did not increase local complications after head and neck free flap reconstruction [21], and in breast reconstruction, free flaps irradiated after transfer have had better flap survival than those transferred into an irradiated field, with comparable complication rates [22]. Third, the site of residual disease should remain accessible to inspection rather than being buried beneath bulk, because close surveillance for at least 5 years is recommended [13]. Whether the ALT island flap satisfies these requirements in practice could not be tested in this patient, who underwent neither re-excision nor radiotherapy and whose surveillance ended at 6 months; they should therefore be read as a proposed rationale rather than a demonstrated outcome. We do not claim that the flap was selected on these grounds prospectively; the case indicates in retrospect that a positive margin accepted intentionally is itself an argument for flap coverage, and that the decision to preserve function and the decision about how to resurface the defect are best made together at the operating table.

The anatomy of this case also influenced flap selection. The ALT island flap is well established for perineoscrotal reconstruction [11,12]; its use here was guided by the distribution of the disease. Thigh involvement in this patient was a medial, perineal extension rather than a separate lesion, so the ipsilateral ALT territory, on the anterolateral thigh, lay well away from the involved skin. Because the penoscrotal, perineal, and medial thigh components formed one continuous defect, a single island flap was sufficient to resurface the entire area, with enough bulk to restore scrotal contour and support the testes and a pedicle long enough to reach the perineum without microvascular anastomosis [11,12]. Split-thickness grafting tolerates the mobile, convex scrotal surface poorly and may contract [9], and scrotal advancement flaps require residual scrotal skin, of which too little remained. Reported experience with the ALT flap after EMPD excision remains confined to small series and isolated reports, to which the present case adds a defect reaching the anal verge resurfaced with a single island flap [11,12].

The donor site, rather than the flap, dominated the postoperative course. A thigh defect wider than approximately 8 cm can rarely be closed primarily without excessive tension, and donor sites left to skin grafting carry greater morbidity, including restricted hip and knee motion from adhesion of the graft to the underlying fascia [23]. Specific to island transfer, the pedicle passes out of the same compartment, so approximating the thigh tightens the tissues around the vessels; trial closure compressed the pedicle here and was abandoned in favor of graft coverage. That decision protected the flap, but the coverage failed. Harvesting the ALT with the fascia lata leaves the vastus lateralis exposed, and a matrix-and-graft construct over bare muscle requires a degree of immobility that is difficult to achieve on the mobile proximal thigh next to the groin. The frog-leg position adopted to protect the perineal inset compounds this, since hip flexion and abduction displace the anterolateral thigh skin over the underlying muscle: the positioning that safeguards the recipient site works against the donor site. Definitive closure required local advancement of the skin and muscle on postoperative day 19. Whether graft failure resulted from shear, delayed incorporation associated with the dermal matrix, or both, cannot be determined from one case, but donor-site coverage after a wide ALT harvest is clearly a reconstructive problem in its own right, for which negative-pressure bolstering or staged closure deserves consideration at the outset.

If an accepted positive margin is justified by the treatment meant to follow, that treatment should be specified before discharge. For intraepidermal disease at a functionally critical site the options are topical imiquimod 5%, photodynamic therapy, and radiotherapy, each achieving local clearance in selected patients but each with substantial recurrence rates [13,24,25]; radiotherapy, delivered at 32–64.8 Gy in 20–30 fractions in reported postoperative series [19], is the usual choice where further surgery is unacceptable and is compatible with a well-vascularized flap in the field. Systemic chemotherapy is used mainly for distant metastatic disease [13]; where disease is invasive or overexpresses HER2, options extend to taxane-based chemotherapy and to trastuzumab, alone or with docetaxel [26]. Here the permanent specimen showed disease confined to the epidermis, placing the residual disease within reach of topical, photodynamic, and radiation treatment; HER2 was not assessed, and would have been required had invasion been found. Selection depends on the depth of invasion and on receptor status, which argues for obtaining both from the resection specimen at the time a positive margin is accepted rather than afterwards. In our patient, medical oncology recommended surveillance, with radiotherapy or systemic therapy to be started on progression, and no modality, dose, or schedule was specified in advance. The accepted margin also needs a surveillance schedule agreed before discharge, with examination at regular intervals for at least 5 years and biopsy of any suspicious area [13], since the accessibility that flap coverage preserves is only useful if someone is looking. Our patient was examined 5 days, 2 weeks, and 1, 3, and 6 months after discharge; no suspicious area was found, and no biopsy was taken.

An accepted positive margin is, finally, a conditional decision whose justification rests on the treatment and surveillance meant to follow. In this patient, these conditions were not met: further treatment was neither specified before discharge nor given, and he stopped attending after the 6-month visit, so the status of the residual disease is unknown. The limitations are therefore substantial. This is a single case with short follow-up that ended while the patient was known to harbor residual disease, and because no further treatment was delivered the report can say nothing about oncologic safety or durable local control; the absence of clinically apparent recurrence at 6 months is not evidence of disease control. Secondary EMPD was not formally excluded, because colonoscopy, cystoscopy, urine cytology, fecal occult blood testing, PSA testing, and immunohistochemistry for CK20 and GCDFP-15 were not performed [14,15]; the retained perianal skin was not biopsied; and sexual function was not assessed. What the case can support is narrower—that a positive margin accepted for functional reasons imposes specific requirements on the reconstruction, which we propose on the basis of published evidence, and that a pedicled ALT island flap is a reasonable option to meet them. Longer follow-up and further reported cases, ideally with documented treatment of the accepted margin, are needed before this reconstructive strategy can be recommended broadly.

## 4. Conclusions

In summary, scrotal EMPD should be suspected in any long-standing genital “eczema” and expected to extend beyond what can be seen. Where anatomic constraints make a positive margin the lesser harm, we propose that this choice should guide the reconstruction toward tissue that can be re-elevated, tolerates irradiation, and leaves residual disease open to inspection. In this patient, no further treatment was delivered and follow-up ended at 6 months; these requirements therefore remain untested, and the oncologic outcome is unknown. The ALT island flap provided stable coverage of a continuous penoscrotal, perineal, and medial thigh defect, and its donor site deserved as much planning as the recipient site.

## Figures and Tables

**Table 1:** Chronological summary of the clinical course. ALT, anterolateral thigh; CEA, carcinoembryonic antigen; CK7, cytokeratin 7; CK20, cytokeratin 20; CT, computed tomography; EMPD, extramammary Paget’s disease; GCDFP-15, gross cystic disease fluid protein 15; MRI, magnetic resonance imaging; and PSA, prostate-specific antigen.

Time	Event
9 years before presentation	Onset of a pruritic erythematous scrotal lesion; treated intermittently as eczema at a local dermatology clinic without improvement
March 2025	Referral to the department of dermatology; punch biopsy suggestive of EMPD; transfer to the department of plastic surgery
April 2025	Repeat punch biopsy confirming EMPD (positive for CEA and CK7; negative for p63 and Melan-A); staging with contrast-enhanced CT, pelvic MRI, and bone scintigraphy showing no nodal, visceral, or distant disease; no palpable inguinal lymph nodes; immunohistochemistry for CK20 and GCDFP-15, colonoscopy, cystoscopy, urine cytology, fecal occult blood testing, and PSA were not performed
May 2025	Wide excision with a 2-cm clinical margin; frozen section showing intraepidermal Paget cells at the perianal margin only; further excision not performed, to preserve the anal sphincter, after intraoperative multidisciplinary consultation; reconstruction with an 18 × 12 cm ALT island flap; donor site covered with acellular dermal matrix and a split-thickness skin graft. Permanent pathology: EMPD confined to the epidermis and adnexal epithelium, without dermal, lymphovascular, or perineural invasion; deep margin and all peripheral margins clear except the perianal margin
Postoperative day 19	Failure of the donor-site graft leaving a 15 × 8 cm defect; local advancement of skin and underlying muscle under local anesthesia
5 days to 3 months after discharge	Outpatient visits at 5 days, 2 weeks, 1 month, and 3 months; reconstructed area and perianal skin stable; no biopsy taken. At 1 month after discharge, the patient visited medical oncology; surveillance was recommended, with radiotherapy or systemic therapy to be started on progression; no modality, regimen, dose, or schedule defined
6 months after discharge	Last outpatient visit; both sites healed with no clinically apparent recurrence; normal defecation, voiding, and gait, unrestricted hip and knee motion; further treatment was not started because surveillance had been recommended and no progression was observed
After 6 months	Lost to follow-up; no further treatment delivered

## Data Availability

The data presented in this study are available from the corresponding author upon reasonable request.

## Abbreviations

The following abbreviations are used in this manuscript:

EMPD	Extramammary Paget’s disease
ALT	anterolateral thigh
CEA	carcinoembryonic antigen
CK7	cytokeratin 7
CK20	cytokeratin 20
CT	computed tomography
GCDFP-15	gross cystic disease fluid protein 15
HER2	human epidermal growth factor receptor 2
MRI	magnetic resonance imaging
PSA	prostate-specific antigen
